# Ultrasound‐Triggered Delivery of Iproplatin from Microbubble‐Conjugated Liposomes

**DOI:** 10.1002/open.202100222

**Published:** 2021-10-27

**Authors:** Richard Browning, Nia Thomas, Laura K. Marsh, Louise R. Tear, Joshua Owen, Eleanor Stride, Nicola J. Farrer

**Affiliations:** ^1^ Chemistry Research Laboratory University of Oxford 12 Mansfield Road Oxford OX1 3TA UK; ^2^ Institute of Biomedical Engineering University of Oxford Oxford OX3 7DQ UK

**Keywords:** cancer, drug delivery, iproplatin, liposome, ultrasound

## Abstract

The Pt^IV^ prodrug iproplatin has been actively loaded into liposomes using a calcium acetate gradient, achieving a 3‐fold enhancement in drug concentration compared to passive loading strategies. A strain‐promoted cycloaddition reaction (azide‐ dibenzocyclooctyne) was used to attach iproplatin‐loaded liposomes L(Pt) to gas‐filled microbubbles (M), forming an ultrasound‐responsive drug delivery vehicle [M−L(Pt)]. Ultrasound‐triggered release of iproplatin from the microbubble‐liposome construct was evaluated in cellulo. Breast cancer (MCF‐7) cells treated with both free iproplatin and iproplatin‐loaded liposome−microbubbles [M−L(Pt)] demonstrated an increase in platinum concentration when exposed to ultrasound. No appreciable platinum uptake was observed in MCF‐7 cells following treatment with L(Pt) only or L(Pt)+ultrasound, suggesting that microbubble‐mediated ultrasonic release of platinum‐based drugs from liposomal carriers enables greater control over drug delivery.

## Graphical abstract

**Actively loading platinum(IV) prodrug** iproplatin into liposomes has been achieved using a calcium acetate gradient. Microbubble‐mediated release and accumulation of the prodrug in MCF7 cancer cells following ultrasound treatment demonstrates that this strategy can enable greater control over drug delivery.

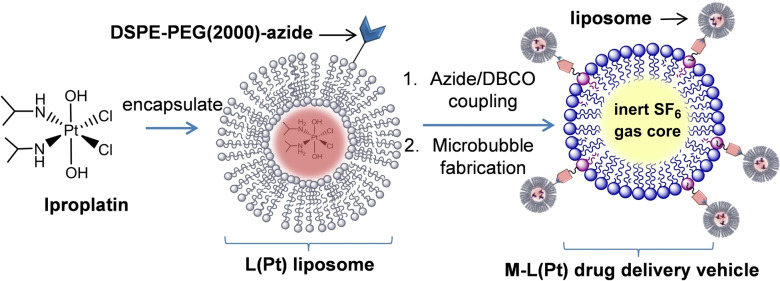

## Introduction

**Scheme 1:**
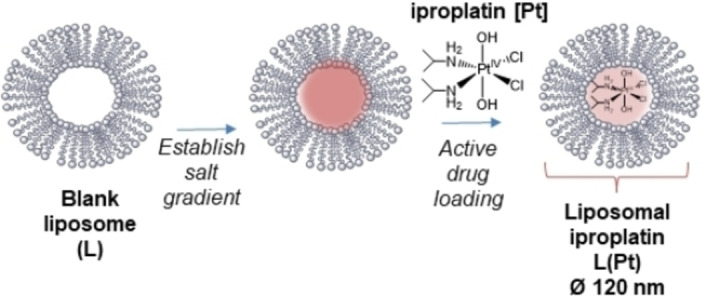
Liposomal encapsulation of platinum (IV) anti‐cancer complex iproplatin [Pt] through active loading to form liposomes L(Pt) which are investigated in this work.

Platinum(II) compounds such as cisplatin are well‐established as highly effective anti‐cancer agents. It is estimated that 50 % of all chemotherapy regimens worldwide include a platinum‐based drug.^[1]^ Platinum(II) drugs are, however, highly reactive in vivo and the side effects of treatment caused by off‐target reactivity are often debilitating and can be so serious that treatment needs to be stopped.^[2]^ Strategies for the controlled delivery of platinum‐based drugs are therefore highly desirable.^[3]^ In comparison to platinum(II) complexes, octahedral platinum(IV) complexes are more kinetically inert due to their 5d^6^ electronic configuration and they typically require reduction to platinum(II) before exerting their anti‐cancer effect.^[4]^ The choice^[5]^ and arrangement^[6]^ of groups in the ligand sphere of the platinum(IV) centre greatly influences both the rate and mechanism of reduction to platinum(II) species as well as the mechanism(s) of anti‐cancer activity. In addition to novel platinum(IV) anti‐cancer complexes currently being developed by ourselves and others,[^7^, ^8^, ^9^, ^10^, ^11^] a number have been clinically evaluated, including satraplatin^[12]^ and iproplatin (*cis,trans,cis*‐dichloridodihydroxidobis(isopropylamine)platinum‐(IV), also known as CHIP and JM9; Scheme 1). Iproplatin, in particular, has been studied in numerous Phase I and II clinical trials.[^13^, ^14^] A Phase III trial for epithelial ovarian cancer concluded that iproplatin would be an acceptable alternative to cisplatin in chemotherapy regimens, since it was comparably effective and demonstrated reduced off‐target toxicity.^[15]^ However, iproplatin still elicits toxic side effects, including myelosuppression and cumulative thrombocytopenia, and as a consequence has failed to gain market approval. Platinum(IV) complexes such as iproplatin are thought to predominantly enter cells through passive diffusion,^[16]^ since they lack the available coordination sites to enable interaction with copper transporters which are partly implicated in the cellular accumulation of platinum(II) complexes, along with anion/cation transporters and passive diffusion.^[17]^ Methods for enhancing their delivery are therefore desirable.

Lipid‐based nanoparticles such as liposomes are particularly attractive drug delivery vehicles due to their biocompatibility.[^18^, ^19^] They have recently been investigated for delivery of platinum(IV) complexes through either encapsulation^[20]^ or membrane anchoring.^[21]^ Several liposomal formulations of cytotoxic drugs are clinically established (e. g., Doxil®)^[22]^ or in clinical development, including the cisplatin‐based Lipoplatin and Nanoplatin™ (Regulon) and LiPlaCis® (Oncology Venture).[^23^, ^24^, ^25^, ^26^]

Liposomal encapsulation can improve drug circulation times and reduce acute toxicity. It can also enhance intracellular delivery, provided the liposomes are in contact with the target cells. Liposomes do not, however, typically improve the selectivity of drug delivery to a target tissue.^[27]^ To address this, multiple strategies are under investigation, including endogenously targeted liposomes (enzymatic digestion,^[28]^ receptor overexpression^[29]^), and formulations that are sensitive to external stimuli to promote drug release (e. g., light,^[30]^ ultrasound (US)^[31]^ and mechanical stress^[32]^). These potentially offer both spatial and temporal control over drug release,^[33]^ thus enabling a smaller overall dose to be administered, and potentially reducing the side effects of treatment.

Microbubbles (MB) – small gas‐filled lipid‐coated bubbles – have been used clinically in medical imaging for several decades.^[34]^ Destruction of MB with focused ultrasound pulses (through a process known as inertial cavitation) creates a number of mechanical effects that can promote drug delivery.^[35]^ Ultrasound and MBs have been used to enhance cellular uptake of both free^[36]^ and liposomally‐encapsulated drugs.[^37^, ^38^] The fluid motion (microstreaming) caused by the oscillation of bubbles in an ultrasound field can also aid extravasation in solid tumours, that is, “pumping“ drugs from the bloodstream across the endothelium, which lines the blood vessels, into the tumour tissue. Microstreaming provides a potential strategy to challenge the high interstitial pressure which is often observed in solid tumours and which inhibits delivery.^[39]^ Inertial cavitation can also trigger drug release from liposomes. For this to be effective, it has been suggested that the MB and liposomes should be less than 40 μm apart.^[40]^ Ultrasound‐mediated drug release from covalently linked MB−liposomal drug vehicles has been shown to enhance drug concentration within a tumour in vivo two‐fold; compared to ultrasound‐mediated release from co‐administered (non‐covalently linked) MB and drug‐loaded liposomes.^[37]^

**Scheme 2:**
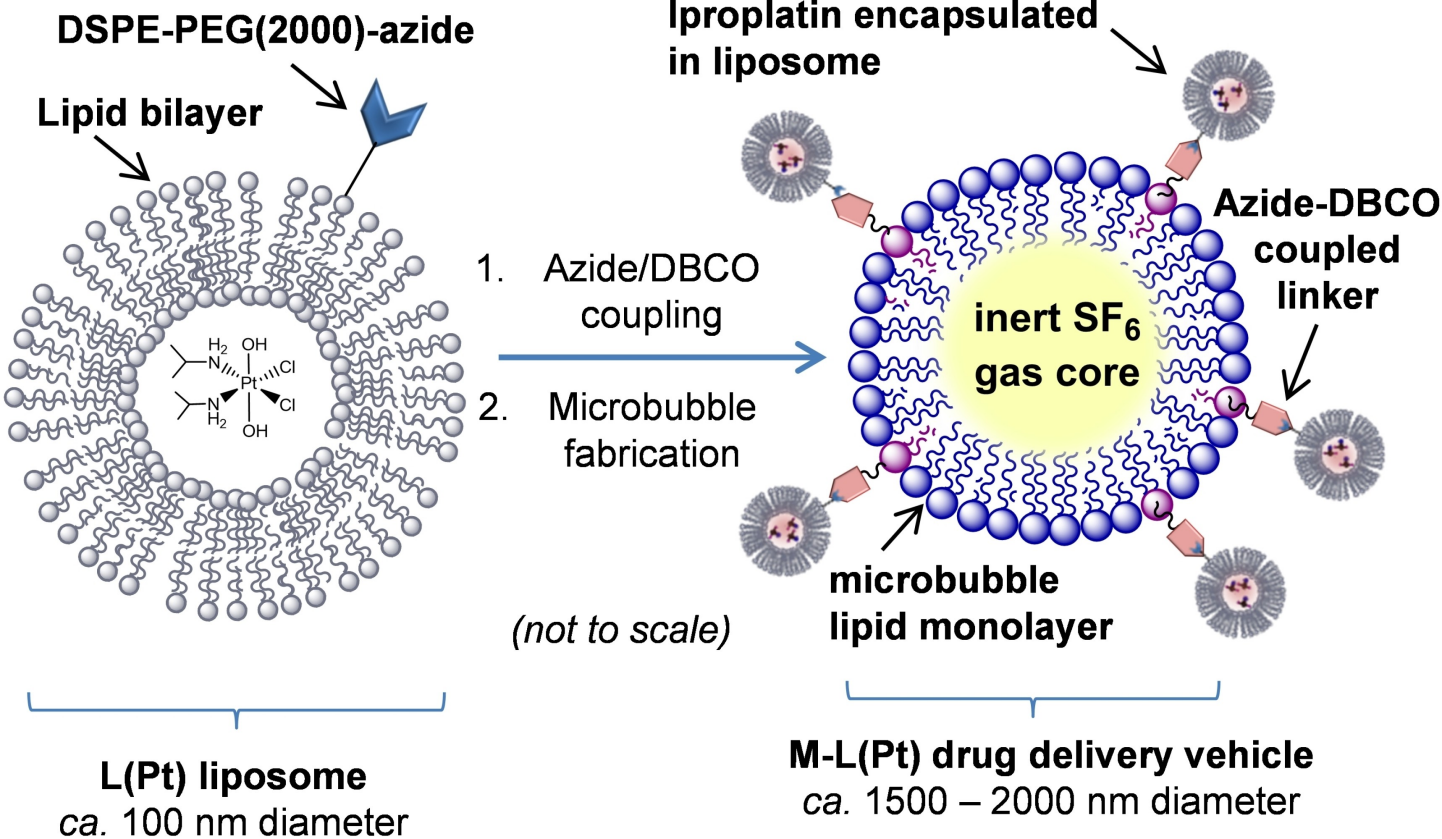
Assembly of the liposome−microbubble drug delivery vehicle MB−L(Pt).

Our aim was to investigate the potential of ultrasound and MB for the delivery of both free and liposomally‐encapsulated platinum(IV) anti‐cancer complexes. We chose iproplatin as a model platinum(IV) complex, since it shows significantly greater aqueous solubility than the platinum(II) complex cisplatin (maximum solubilities of 44 mm and 7 mm respectively),^[41]^ making it potentially amenable to liposomal encapsulation at a higher drug concentration. Here we report our investigations into strategies for the liposomal loading of iproplatin (designated “Pt”, Scheme 1), to form liposomal iproplatin “L(Pt)” and the assembly of these drug‐filled liposomes into microbubble−liposome drug delivery vehicles “MB−L(Pt)” (Scheme 2). We also report our findings from preliminary investigations into the ultrasound‐mediated release and cellular uptake of iproplatin from the drug delivery vehicle MB−L(Pt).

Although biotin‐(strept)avidin couplings are relatively robust, they can be immunogenic in humans and harder to translate.[^50^, ^51^] Maleimide−thiol linkages have been used both to introduce targeting ligands to MB^[52]^ and to couple MB to liposomes.^[46]^ However, maleimide−thiol linkages have the potential to cleave at physiological pH, which may result in liposome shedding.^[53]^ Click chemistry is biorthogonal,^[54]^ produces stable products^[55]^ and is therefore a highly promising strategy for labelling MB.^[56]^

## Results and Discussion

### Platinum (IV) Prodrug Synthesis

The platinum(IV) prodrugs iproplatin^[42]^ and *trans*,*trans*,*trans*‐[Pt(N_3_)_2_(OH)_2_(py)_2_]^[43]^ were synthesised as previously reported and purified by mass‐directed HPLC (see Supporting Information).

### Liposomal Formation and Iproplatin Loading

We investigated the liposomal loading of iproplatin using both passive and active (remote)^[44]^ drug loading strategies, where the active loading strategy treated iproplatin as a weak acid.^[45]^

For both passive and active loading, liposomes were formed by sonication and then sequential extrusion (through 400 nm diameter (Ø), then 200 nm Ø filters). We used an established liposomal composition which has previously demonstrated promise for microbubble‐mediated ultrasonic drug release (molar ratios): DPPC (54); cholesterol (36); DSPE−PEG(2000) (10),^[46]^ where DPPC is 1,2‐dipalmitoyl‐sn‐glycero‐3‐phosphocholine and DSPE−PEG(2000) is 1,2‐distearoyl‐sn‐glycero‐3‐phosphoethanolamine‐N‐[methoxy(polyethylene glycol)‐2000]) (Figure S1). Inclusion of cholesterol increases membrane rigidity and decreases the permeability of the liposome membrane, whilst polyethylene glycol (PEG) adds a steric barrier, increasing circulation times by stealthing the liposomes from immune detection.^[47]^ A small amount (1 mmol %) of fluorescent DiI dye was also added to the liposome formulation to facilitate both liposome visualisation and purification. Passive loading was undertaken by forming liposomes in a saturated (30 mg mL^−1^) solution of iproplatin at 50 °C. Active loading involved forming blank liposomes in 120 mm calcium acetate solution, before establishing a salt concentration gradient, through several rounds of dialysis with 120 mm sodium sulphate solution. This was followed by addition of iproplatin (to a final concentration of 27 mm) to the blank liposome solution, followed by incubation at 55 °C for 1 h. Following encapsulation, unencapsulated iproplatin was removed by size‐exclusion gel‐filtration chromatography (Sephadex).^[45]^

The stability of iproplatin in the presence of the (unextruded) lipid formulation was confirmed through both ^195^Pt and ^31^P NMR spectroscopy; no changes were observed, with monitoring for a week following incubation, at ambient temperature (Figures S2 and S3). Since drug loading has been reported to be improved by freeze‐thawing (FT) and inclusion of sucrose, these were also investigated during liposome fabrication.^[44]^

### Liposome Analysis

Following removal of unencapsulated drug (Sephadex), liposome samples were analysed for both size and platinum concentration. Dynamic light scattering (DLS) was used to measure the diameter (Ø) and polydispersity Index (PdI) of the liposomes; values for liposomes loaded by method **1** (Table 1) (with extrusion through 400 nm Ø filters followed by 200 nm Ø filters) were of average Ø=169 nm and PdI=0.065, where PdI <0.1 indicates a monodisperse solution (Figure S4); liposomes fabricated and loaded through the same extrusion protocol by methods **2** and **3** gave similar data. Platinum concentration (equivalent to iproplatin concentration) and phosphorus concentration (due to phosphate headgroups of DPPC and DSPE−PEG(2000)) was determined by chemical digestion of the samples (conc. HNO_3_/HCl, H_2_O_2_, heat) followed by ICP‐MS analysis. This enabled both the liposome concentration and drug:lipid (d:l) ratio to be determined. The results for the different loading methods are given in Table 1.

**Table 1:** Results of liposomal loading of iproplatin using different loading strategies; reported values for commercial doxorubicin formulation are included for comparison.^[19]^ FT=5 x freeze‐thaw cycles were completed following hydration of the solution before extrusion. Final concentrations of lipid and drug are back‐calculated from ^31^P and ^195^Pt content as determined by ICP‐MS.

Loading method	c(drug) [mg mL^−1^]	c(drug) [mm]	Pt : P ratio	c(lipid) [mg mL^−1^]	Drug/lipid ratio [mg mg^−1^]
**1** – [Pt] (passive, sucrose FT)	0.18	0.43	0.39	8.8	0.02
**2** – [Pt] (120 mm [Ca(OAc)_2_])	0.50	1.20	0.59	16.4	0.03
**3** – [Pt] (120 mm [Ca(OAc)_2_], FT)	0.55	1.31	0.75	14.1	0.04
Doxorubicin in Doxil®	2.0	3.5	N.A.	16.0	0.13

The calcium acetate method (Figure S5) which included a freeze‐thaw step (Method **3**)[^45^, ^48^] enabled the highest concentration of iproplatin to be liposomally loaded, with a Pt : P ratio of 0.75. Method **3** resulted in a drug:lipid ratio of 0.04 which is twice as high as the loading achieved under passive loading conditions, although still falling short of the concentration achieved for the commercially available liposomal formulation Doxil® (0.125) which contains the anthracycline doxorubicin.^[19]^ The high drug loading for doxorubicin is partly attributed to the “stacking” of the planar compound within the liposomes.^[49]^ The final concentration of iproplatin in the liposome suspension was 1.3 mm. Since the starting concentration of iproplatin was 27 mm, this corresponded to a 5 % encapsulation efficiency. To briefly investigate the importance of the ligands on encapsulation, method **3** was also employed to liposomally encapsulate the platinum(IV) complex *trans*,*trans*,*trans*‐[Pt(N_3_)_2_(OH)_2_(py)_2_]^[43]^ which we have previously evaluated previously as a light‐activated prodrug. This compound has no ionisable amine protons, and showed significantly (4 ‐fold) lower drug loading than iproplatin, consistent with the active loading hypothesis for iproplatin outlined in Figure S5.

### Leakage of Iproplatin from Liposomes

The rate of iproplatin leakage from liposomes which had been prepared by loading method **3** was investigated by ICP‐MS. Following preparation and filtration (Sephadex), L(Pt) liposomal solutions were stored at 4 °C. Unencapsulated iproplatin was removed at regular intervals by filtration (Sephadex), with 70 μL aliquots taken for analysis by ICP‐MS (Figure S6). These data indicated a relatively rapid leakage of iproplatin with approximately 19 % iproplatin remaining encapsulated after 6 d; this is consistent with the observation that gradual passive liposomal leakage can be observed for small hydrophilic drugs.[^45^, ^48^]

### Assembly of Iproplatin‐Loaded Microbubble−Liposome Vehicles MB−L(Pt)

We investigated the use of strain‐promoted azide−alkyne (SPAAC) “click” chemistry between azido‐ (N_3_) and dibenzocyclooctyne‐functionalised (DBCO) lipids to covalently assemble MB−L(Pt) drug delivery vehicles_._ To minimise the potential for membrane fusion^[57]^ and since SF_6_‐filled microbubbles have a half‐life on the order of minutes,^[58]^ the click reaction was conducted prior to MB fabrication.^[59]^ To assemble the MB−L(Pt) vehicles, iproplatin‐loaded liposomes were formulated according to method **3**, but with DSPE−PEG(2000) substituted for an equimolar amount of DSPE−PEG(2000)‐N_3_, that is, molar ratios of DPPC (54); cholesterol (36); DSPE−PEG(2000)‐N_3_ (10). The click reaction between L(Pt)‐N_3_ liposomes and DBCO (18 : 1 dibenzocyclooctyl PE) lipid was carried out immediately after liposome fabrication and purification, by addition of the liposome solution to a mixture of DPPC, DSPE−mPEG(2000) and DSPE−PEG(2000)‐DBCO (82 : 9 : 9 molar ratio) which had been rehydrated with an 8 : 1 : 1 mixture of phosphate‐buffered saline (PBS), propylene glycol and glycerine (see Supporting Information). The time to completion for the click reaction was anticipated to depend on the components; although the reaction of DBCO‐functionalised MB with azido‐containing compounds has been reported to complete within 5 min,^[60]^ the reaction between a DBCO‐containing compound and an azido‐functionalised cellular surface component has been reported to continue to progress over a 30 min period.^[61]^ We allowed the click reaction to proceed overnight before microbubble manufacture. SF_6_‐filled microbubbles were then fabricated by replacing the headspace of the vial with SF_6_, followed by agitation with a capsule mixer (Scheme 1).[^46^, ^62^] The MB−L(Pt) vehicles were centrifuged and washed twice with PBS to remove unbound liposomes and unencapsulated iproplatin, before being imaged by microscopy and counted using image analysis software, giving a final concentration of 3.35 (±1.75)×10^8^ MB mL^−1^ and a MB−L(Pt) diameter of 1.81±0.04 μm (Figure S7).

### Ultrasound and Microbubble‐Mediated Cellular Accumulation of Free Iproplatin

**Figure 1:**
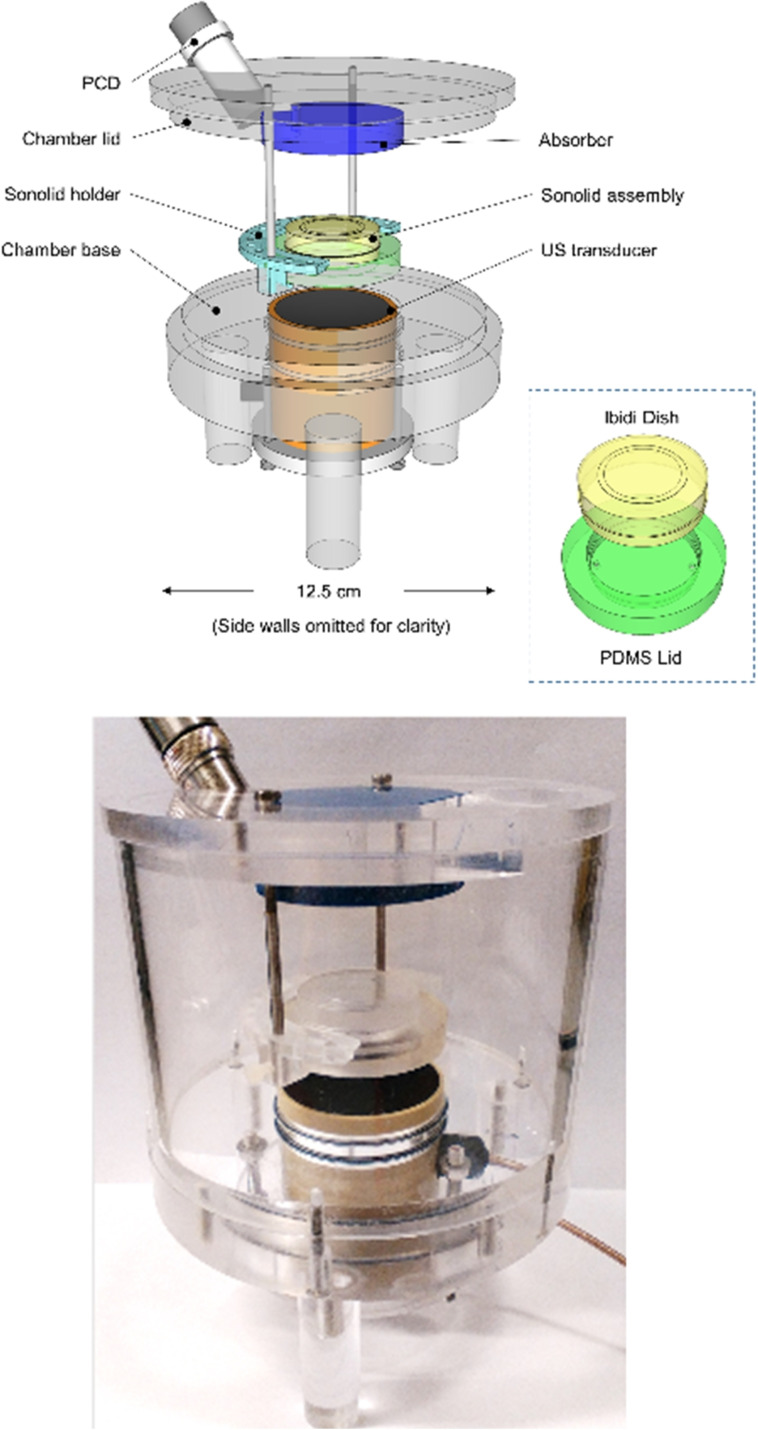
Ultrasound SAT2 device for cellular experiments: schematic (top) and photo (bottom).^[63]^ Ultrasound is applied to the Ibidi dish from below.

We explored the effect of ultrasound and microbubbles on the cellular accumulation of free iproplatin, as a precursor to investigating release and uptake of iproplatin from the MB−L(Pt) delivery vehicle. We wanted to assess accumulation of iproplatin without complications due to cell death and lysis. The MCF7 (breast cancer) cell line was selected due to the relatively modest IC_50_ value of iproplatin in this cell line (254.6 μm, MTT assay, Figure S8) since our focus was on accumulation rather than cell kill. The drug‐cell contact and post‐irradiation exposure times were also kept short. Experiments were conducted using a static ultrasound device (SAT2) (Figure 1). Cells were seeded into Ibidi dishes and a solution of iproplatin with either PBS only, or PBS plus a microbubble solution was mixed and added to the cells. This was followed by irradiation or sham irradiation of the cells for 3 min. Following treatment with ultrasound, the cells were incubated for 60 min, then washed six times with PBS to remove free iproplatin.

Cells were lysed by addition of DMSO, and cellular uptake of free iproplatin was assessed by ICP‐MS (Table 2). It was determined by analysing the cell extract by LCMS that the concentration of unmetabolised iproplatin was below the detection limit of the technique (Figure S9). ICP‐MS data demonstrated that the cellular uptake of iproplatin was enhanced approximately 3‐fold, by both the use of ultrasound only, and the use of ultrasound and microbubbles. Microbubbles and iproplatin – without ultrasound – did not result in a significant enhancement in cellular uptake of iproplatin. The mechanism by which the microbubbles enhance cellular accumulation of iproplatin from solution could involve formation of membrane pores or endocytosis.^[64]^

**Table 2:** Effect of ultrasound and microbubbles on the cellular accumulation of unencapsulated iproplatin [Pt] in MCF7 cells. Ultrasound parameters (centre frequency: 1 MHz, acoustic pressure: 148 kPa (peak‐to‐peak), pulse repetition frequency: 100 Hz, duty cycle: 30 %, pulse length: 3000 cycles, exposure time: 180 s).

Sample	ICP‐MS ^194^Pt in cells [μg g^−1^]
**1**. MB+Pt+US	0.33
**2**. MB+Pt	0.10
**3**. Pt+US	0.37
**4**. Pt	0.08

### Ultrasound‐Mediated Iproplatin Release from L(Pt) and MB−L(Pt) and Cellular Accumulation

The ultrasound‐mediated release and cellular accumulation of iproplatin from both L(Pt) and from MB−L(Pt) was investigated in MCF7 cells. As before, cellular uptake of iproplatin was assessed by ICP‐MS (Table 3). To enable comparison with the free iproplatin experiments, all US parameters were kept the same.

**Table 3:** Effect of ultrasound (US) on iproplatin accumulation in MCF7 cells from liposomes (L(Pt) and microbubble−liposome delivery vehicle MB−L(Pt). Ultrasound parameters: centre frequency: 1 MHz, acoustic pressure: 148 kPa (peak to peak), pulse repetition frequency: 100 Hz, duty cycle: 30 %, pulse length: 3000 cycles, exposure time: 180 s.

Sample	ICP‐MS ^194^Pt in cells [μg g^−1^]
**1**. MB−L(Pt)+US	0.38
**2**. MB−L(Pt)−US	0.27
**3**. L(Pt)+US	<0.002

For ultrasound‐mediated release from L(Pt) only, the cellular concentration of platinum was below the ICP‐MS detection limit. This confirmed both minimal liposomal iproplatin leakage over the time course of the experiment, and the weak echogenicity of liposomes under these ultrasound conditions.

Platinum uptake was also observed from the MB−L(Pt) construct in the absence of ultrasound, which may be due to non‐specific adherence of the microbubbles in the MB−L(Pt) vehicle to the cells, and/or iproplatin release during assembly of the MB−L(Pt) drug delivery vehicle. Platinum accumulation was also observed from the vehicle following the application of ultrasound. Whilst the precise mechanism of ultrasound‐mediated release from the construct requires further investigation, we suggest that iproplatin uptake from liposomes may either involve liposome destruction and uptake of free iproplatin (Figure 1), or microbubble‐mediated fusion of the liposomal membrane with cellular membrane through a pinocytotic mechanism. The mechanism is likely to be influenced by both microbubble shell composition and the acoustic parameters.^[65]^ To confirm the stability of iproplatin itself to ultrasound, ^1^H NMR spectral and HPLC analysis of a 3 mm solution of iproplatin in D_2_O recorded under sham irradiation and following exposure to ultrasound (20 kHz output frequency, 3 min, 30 % amplitude) confirmed that iproplatin remains chemically unchanged under ultrasound irradiation and is therefore anticipated to retain its cytotoxicity following ultrasound release. Although no literature reports of the precise nature of the DNA lesions caused by iproplatin could be found, the mechanisms of action and comparison of DNA lesions formed following either conventional cellular uptake, or following ultrasound‐mediated delivery are of interest and will form part of our ongoing research.

## Conclusions

Calcium acetate active loading enabled a 3‐fold higher concentration of iproplatin to be encapsulated within liposomes in comparison to passive loading. The calcium acetate loading strategy is thought to improve loading through deprotonation of the iproplatin amine ligand, and therefore may be suitable for enhancing liposomal loading of other platinum(IV) complexes which incorporate a protonated amine group. Gradual leakage of iproplatin from the liposomes occurred in solution. Work is therefore underway to improve liposomal retention of the actively‐loaded platinum drug.

Azido−DBCO click chemistry was used for linking microbubbles and liposomes, assembling a microbubble−liposome drug delivery vehicle (MB−L(Pt)). Whilst ultrasound irradiation of iproplatin‐filled liposomes alone did not result in detectable platinum cellular uptake, cellular uptake of platinum from the MB−L(Pt) delivery vehicle was observed both in the absence and presence of ultrasound. Platinum uptake in the absence of ultrasound could be caused by a number of factors, including the use of a static cellular model.

To conclude, our preliminary investigations have identified both the potential for, and the current limitations of, our chosen ultrasound‐mediated delivery strategy for platinum(IV) anti‐cancer agents. The modular nature of the MB−L(Pt) delivery vehicle provides the potential for the controlled release of encapsulated platinum drugs in the immediate vicinity of the tumour, which has the potential to significantly improve delivery and reduce off‐target activity of platinum(IV) prodrugs.

## Experimental Section

Iproplatin was synthesised by adaptation of literature reports, details and characterisation data are given in the Supporting Information.^[42]^ For additional materials, methods and ultrasound parameters, see the Supporting Information.

### Optimal Liposome Loading Method: Method 3

DPPC (850355P) (72.5 mg, 0.099 mmol), cholesterol (700000P) (24.8 mg, 0.064 mmol) and DSPE−mPEG(2000) (880120P, 50.4 mg, 0.018 mmol) in molar ratios of 54 : 36 : 10 respectively were dissolved in chloroform (2 mL) in a 500 mL RBF and DiI (50 μL of 2 mg mL^−1^ stock solution in chloroform) was added. The solution was swirled to dissolution, and the solvent removed by rotary evaporation; gradually reducing the vacuum (at 60 °C) with fast rotation to give a thin, even film over the bottom third of the flask. The flask was placed under high vacuum overnight, before replacing the headspace with nitrogen. The lipid film was hydrated with minimal (5 mL) 120 mm ^[3]^ aqueous calcium acetate solution under rotary evaporation (at 60 °C) for 20 min followed by vortexing, until the solution became transparent.^[4]^

2 mL of the stock lipid solution was taken through 5 freeze‐thaw cycles between liquid N_2_ and the water bath (55 °C) with 10 s of vortexing as the solution warmed up. The solution was left at each temperature extreme for 5 min. 1.2 mL of the lipid solution was then taken from the water bath at (60 °C), transferred into a 1 mL extrusion syringe, and set on an extrusion block and syringe/filter apparatus which had been pre‐heated to 60 °C. The solution was extruded 23 times through a 400 nm filter. The equipment was rinsed with MeOH followed by 120 mm aqueous calcium acetate solution. The solution was then extruded 23 times through 200 nm filter and cooled to 4 °C for 10 min.

*Gradient Establishment*: 0.95 mL of the liposome solution was recovered and transferred to a pre‐prepared Float‐a‐Lyser G2 0.5–1 kDa 1 mL dialysis tube, and the solution dialysed against 200 mL of 120 mm sodium sulphate (Na_2_SO_4_) solution (buffer preheated to 40 °C) for 1 hr. This was repeated twice more, using 200 mL of 120 mm sodium sulphate (Na_2_SO_4_) solution (buffer preheated to 40 °C). A fourth cycle of dialysis against 400 mL 120 mm sodium sulphate (Na_2_SO_4_) solution was conducted at ambient temperature for 12 h.

*Drug Loading*: The liposome solution (0.95 mL) was transferred to a glass vial and 14 mg iproplatin were added. The solution was stirred gently at 55 °C for 1 h. The solution was cooled to 4 °C and then columned on Sephadex columns (pre‐equilibrated with 120 mm sodium sulphate (Na_2_SO_4_) solution) to remove unencapsulated drug. The pink band corresponding to the liposomes was collected; unencapsulated iproplatin was observed as a yellow band which followed the liposome band. A 200 μL aliquot of the sample was removed for digestion and analysis by ICP‐MS.

## Conflict of interest

The authors declare no conflict of interest.

## Supporting information

As a service to our authors and readers, this journal provides supporting information supplied by the authors. Such materials are peer reviewed and may be re‐organized for online delivery, but are not copy‐edited or typeset. Technical support issues arising from supporting information (other than missing files) should be addressed to the authors.

Supporting Information
