# Supplementary material for: Ultrasound‐Triggered Delivery of Iproplatin from Microbubble‐Conjugated Liposomes
Source: ChemistryOpen. 2021 Oct 27;10(12):1170–6. doi: 10.1002/open.202100222 (PMC8634767; doi:10.1002/open.202100222)
Supplement: Supplementary file 1 — Supporting Information [file OPEN-10-1170-s001.pdf]

ChemistryOpen

Supporting Information

Ultrasound-Triggered Delivery of Iproplatin from Microbubble-Conjugated Liposomes

Richard Browning, Nia Thomas, Laura K. Marsh, Louise R. Tear, Joshua Owen, Eleanor Stride, and Nicola J. Farrer*

Contents

Materials and methods 1
Materials 1
Chemical methods 2
Synthesis of iproplatin 2
Liposome loading protocols 4
Concentration determination of iproplatin in liposomes 6
Liposomal ICP-MS digestion protocol 6
General microbubble (MB) Synthesis 7
Production of coupled MB-L(Pt) 7
Biological methods 8
General Ultrasound Methods 8
Ultrasound platinum cellular uptake experiments 8
Ultrasound and microbubble-mediated cellular accumulation of free iproplatin 8
Ultrasound-mediated iproplatin release from L(Pt) and MB-L(Pt) and cellular accumulation 9
Structures of lipids and cholesterol used 10
Stability of iproplatin in the presence of lipids 11
Stability of iproplatin to ultrasound 13
Iproplatin Calibration Curve for LCMS 19

Materials and methods

Note – DiI is light-sensitive. All solutions which included DiI were kept wrapped in foil to exclude light.

Materials

$K_2[PtCl_4]$ was purchased from Precious Metals Online. HPLC-grade solvents and Millipore-filtered H_2O were used for the preparation of compounds and purification by HPLC. DiI was purchased from Abcam PLC. Lipids and cholesterol were purchased from Avanti Polar directly or *via* Stratech Scientific Ltd. and used as received. All other chemical reagents were reagent grade and were purchased from standard commercial vendors (*e.g.* Sigma-Aldrich or Alfa Aesar). (IM) indicates use of a syringe filter (pore size 0.2 µM). Pre-loaded Sephadex G-25 columns (PD midi-trap™ G-25, GE Healthcare) were purchased from VWR and were used to remove unencapsulated drug following liposomal loading. Liposomes were manufactured by extrusion using an *Avanti* Mini-Extruder equipped with two 1000 µL syringes set in a heating block and used as specified in the manufacturer protocol.[1]

Chemical methods

NMR spectroscopy. Spectra were acquired at 298 K unless otherwise stated, and processed using Topspin 3.2. Chemical shift (δ) values are given in parts per million and are referenced to residual solvent unless otherwise stated, *J* values are quoted in Hz. ^{1}H NMR spectra: were acquired on a Bruker AVIIIHD 500 MHz (500.13 MHz) equipped with a 5mm z-gradient broadband X-^{19}F/^{1}H BBFO SMART probe or a Bruker AVIIIHD 400 nanobay (400.17 MHz). ^{195}Pt NMR and ^{31}P NMR spectra: were acquired on a Bruker AVII 500 MHz spectrometer equipped with a z-gradient triple resonance inverse ^{1}H/^{19}F(^{13}C) TXI probe. ^{195}Pt NMR chemical shifts were externally referenced to K_2PtCl_6 in 1.5 mM HCl in D_2O (δ 0 ppm).

Mass Spectrometry. Low resolution ESI-MS were obtained with a Waters Micromass LCT Premier XE spectrometer. HRMS were obtained with a Thermofisher Exactive Plus with a Waters Acuity UPLC system. MS/MS experiments. Performed on an Acuity UPLC in flow injection analysis mode, equipped with a Waters Xevo G25 QTOF. MS data were processed using MassLynx 4.0.

HPLC. HPLC was performed with a Waters Autopurification system. Prep-HPLC used a Waters X-Bridge OBD semi-prep column (5 µm, 19 mm x 50 mm), with an injection loop of 1 ml, eluting with H_2O+0.1% NH_4OH (pH 9)/MeCN +0.1% NH_4OH. Samples (in H_2O/MeCN) were filtered (IM, nylon) and injected in 750 µL aliquots, with mass-directed purification with an ACQUITY QDa performance mass spectrometer. Analytical HPLC used the same solvents and a Waters X-Bridge OBD column (5µm, 4.6 mm x 50 mm) and an injection loop of 0.02 ml. UV-visible absorption spectra were obtained with a T60U Spectrometer PG Instruments Ltd using UVWin Software, or the Waters HPLC.

ICP-MS: Elemental Pt and P concentrations were determined using a Perkin Elmer 6100DRC ICP-MS instrument by Dr. Phil Holdship in the Department of Earth Sciences, University of Oxford.

Dynamic Light scattering (DLS): Liposomes were sized by dynamic light scattering using a Malvern Zetasizer Nano ZS (Malvern Panalytical, Malvern, UK). Liposomes were diluted in either water or 120 mM sodium sulphate depending upon method of production, and loaded into the machine in disposable cuvettes. A standard operating protocol (SOP) was set up in the associated Zetasizer software using the appropriate diluent and a literature value of refractive index for the liposome (1.45).[1] All other settings were default as programmed by the manufacturer. Size and polydispersity index (PDI) values were taken from intensity vs size plots.

Microscopy: Brightfield microscope images were captured using a Leica microscope (Leica Microsystems GmbH, Wetzlar, Germany), with a QImaging digital camera (MicroPublisher 3.3 RTV, QImaging, Surrey, Canada). The processing software was LivingImage v8 (Media Cybernetics, Inc., Rockville, MD, USA). For fluorescence imaging, a Zeiss Laser Scanning Microscope 710 (LSM 710) (Carl Zeiss Microscopy GmbH, Jena, Germany) was used. The processing software was ZEN (Zeiss). For samples containing liposomes, wavelengths were selected for DiI detection (λ_{ex} = 549 nm, λ_{ex} = 565 nm).

Synthesis of iproplatin

The synthesis of iproplatin was adapted from various literature sources[2,3] and is summarised below.

***Cis*-[$PtI_2(H_2NPr^i)_2$]:**[3] A solution of K_2PtCl_4 (0.5 g, 2.4 mmol) and KI (1.94 g, 4.8 eq., 11.7 mmol) in H_2O (25 ml) was stirred in the dark at room temperature for 1 h, before isopropylamine (0.2 ml, 2 eq.,

4.8 mmol) was added dropwise over a 10 minute period. The solution was then stirred for a further 4 h. The precipitate was collected and washed with cold water, methanol and diethyl ether, giving the title compound as an orange solid (442 mg, 0.8 mmol, 73%). ^{1}H-NMR (400 MHz, acetone-d_6) δ: 4.38 (4H, br, NH_2), 3.57 (2H, sept, CH), 1.37 (12H, d, CH_3). ^{195}Pt-NMR (500 MHz, acetone-d_6) δ: –3346.

***Cis*-[PtCl$_2$(H$_2$NPr^i)$_2$]:** *cis*-[PtI$_2$(H$_2$NPr^i)$_2$] (442 mg, 0.8 mmol) was suspended in H_2O (30 ml) and $AgNO_3$ (250.g, 1.9 eq., 1.48mmol) was added. The solution was stirred in the dark overnight. AgI was removed by filtration (celite) and 4 eq. NaCl (180 mg, 4 eq., 3.08 mmol) added to the filtrate. The solution was stirred at 40°C overnight then cooled to 4°C which resulted in the formation of a yellow precipitate which was isolated by filtration. The solid was washed with cold water, methanol and diethyl ether to give the title compound as a yellow solid (143 mg, 0.38 mmol, 47%). ^{1}H-NMR (400 MHz, DMSO-d_6) δ: 4.91 – 4.65 (4H, br, NH_2), 3.11 (2H, sept, CH), 1.22 (12H, d, CH_3) ppm. ^{195}Pt-NMR (500 MHz, DMSO-d_6) δ: – 2215.

***Cis,trans,cis*-[PtCl$_2$(OH)$_2$(H$_2$NPr^i))$_2$] (iproplatin):**[2] *cis*-[PtCl$_2$(H$_2$NPr^i)$_2$] (143 mg, 0.38 mmol) was suspended in H_2O (5 ml) and H_2O_2 (30% w/w, 0.7 ml) was added dropwise. The solution was stirred at 50 ^{o}C overnight. The volume of the filtrate was reduced *in vacuo* to dryness. The product was purified by mass-directed HPLC (triggering on M= 441 *m/z*) to give the title compound as a yellow solid (60 mg, 0.15 mmol, 53%). ^{1}H NMR 400 MHz, D_2O) δ: 3.27 (sept, 2H, CH), 1.30 (d, 12H, CH_3), 0.42 (br, 2H, OH). CHN calc (%): 17.26, 4.83, 6.72; found (%): 17.12, 4.91, 6.72. ^{195}Pt NMR (500 MHz, D_2O) δ: 938.

ESI-MS (MeOH) key species *m/z*; 419.10 ([M+H]$^+$, *weak*), 441.03 ([M+Na]$^+$; calc. $C_6H_{20}Cl_2N_2NaO_2Pt$, 441.04); 837.12 ([2M+H]$^+$ calc. $C_{12}H_{41}Cl_4N_4O_4Pt_2$, 837.11).

Liposome loading protocols

Method 1

DPPC (850355P) (8.83 mg, 0.012 mmol), cholesterol (700000P) (3.1 mg, 0.008 mmol) and DSPE-PEG(2000) (880120P, 6.25 mg, 0.002 mmol) of molar ratios of 54:36:10 respectively were dissolved in chloroform (2 mL) in a 250 mL RBF and DiI (50 µL of 2mg.mL^{-1} stock solution in chloroform) was added. The solution was swirled to dissolution, and the solvent removed by rotary evaporation; gradually reducing the vacuum (at 40 °C) with fast rotation to give a thin, even film over the bottom third of the flask. The flask was placed under high vacuum overnight, before replacing the headspace with nitrogen. The lipid film was hydrated with 2.5 mL of an aqueous solution (containing 15 mg iproplatin in a 10% sucrose solution) at 60 °C under rotary evaporation for 60 min, followed by 1 min sonication.

The solution was taken through 5 freeze-thaw cycles between liquid N_2 and the water bath (55 °C) with 30 s of vortexing as the solution warmed up. The solution was left at each temperature extreme for 5 min.

1.2 mL of the lipid solution was taken from the water bath at (60 °C), transferred into a 1 mL extrusion syringe, and set on an extrusion block and syringe/filter apparatus which had been pre-heated to 60 °C. The solution was extruded with 23 passes through a 400 nm filter. The equipment was rinsed with MeOH followed by 10 % sucrose solution. The solution was then extruded again: 23 times through a 200 nm filter and cooled to 4 °C for 10 min to give 0.45 mL of the liposome solution. 0.55 mL of 10 % sucrose was added to make the volume up to 1 mL, and the solution was columned on Sephadex columns (pre-equilibrated with 10 % sucrose solution) to remove unencapsulated drug. The pink band corresponding to the liposomes was collected; unencapsulated iproplatin was observed as a yellow band which followed the liposome band.

Method 2

DPPC (850355P) (72.5 mg, 0.099 mmol), cholesterol (700000P) (24.8 mg, 0.064 mmol) and DSPE-mPEG(2000) (880120P, 50.4 mg, 0.018 mmol) in molar ratios of 54:36:10 respectively were dissolved in chloroform (2 mL) in a 500 mL RBF and DiI (50 µL of 2 mg.mL^{-1} stock solution in chloroform) was added. The solution was swirled to dissolution, and the solvent removed by rotary evaporation; gradually reducing the vacuum (at 60 °C) with fast rotation to give a thin, even film over the bottom third of the flask. The flask was placed under high vacuum overnight, before replacing the headspace with nitrogen. The lipid film was hydrated with minimal (5 mL) 120 mM[3] aqueous calcium acetate solution under rotary evaporation (at 60 °C) for 20 min followed by vortexing, until the solution became transparent.[4]

1.2 mL of the stock lipid solution was then taken from the water bath at (60 °C), transferred into a 1 mL extrusion syringe, and set on an extrusion block and syringe/filter apparatus which had been pre-heated to 60 °C. The solution was extruded 23 times through a 400 nm filter. The equipment was rinsed with MeOH followed by 120 mM aqueous calcium acetate solution. The solution was then extruded 23 times through 200 nm filter and cooled to 4 °C for 10 min.

Gradient establishment: 0.95 mL of the liposome solution was recovered and transferred to a pre-prepared Float-a-Lyser G2 0.5-1 kDa 1 mL dialysis tube, and the solution dialysed against 200 mL of 120 mM sodium sulphate (Na_2SO_4) solution (buffer preheated to 40 °C) for 1 hr. This was repeated twice more, using 200 mL of 120 mM sodium sulphate (Na_2SO_4) solution (buffer preheated to 40 °C).

A fourth cycle of dialysis against 400 mL 120 mM sodium sulphate (Na_2SO_4) solution was conducted at ambient temperature for 12 h.

Drug loading: The liposome solution (0.95 mL) was transferred to a glass vial and 14 mg iproplatin was added. The solution was stirred gently at 55 ^oC for 1h. The solution was cooled to 4^oC and then columned on Sephadex columns (pre-equilibrated with 120 mM sodium sulphate (Na_2SO_4) solution) to remove unencapsulated drug. The pink band corresponding to the liposomes was collected; unencapsulated iproplatin was observed as a yellow band which followed the liposome band.

Method 3 (with freeze-thaw step)
DPPC (850355P) (72.5 mg, 0.099 mmol), cholesterol (700000P) (24.8 mg, 0.064 mmol) and DSPE-mPEG(2000) (880120P, 50.4 mg, 0.018 mmol) in molar ratios of 54:36:10 respectively were dissolved in chloroform (2 mL) in a 500 mL RBF and DiI (50 µL of 2mg.mL^{-1} stock solution in chloroform) was added. The solution was swirled to dissolution, and the solvent removed by rotary evaporation; gradually reducing the vacuum (at 60 ^oC) with fast rotation to give a thin, even film over the bottom third of the flask. The flask was placed under high vacuum overnight, before replacing the headspace with nitrogen. The lipid film was hydrated with minimal (5 mL) 120 mM[3] aqueous calcium acetate solution under rotary evaporation (at 60 ^oC) for 20 min followed by vortexing, until the solution became transparent.[4]

2 mL of the stock lipid solution was taken through 5 freeze-thaw cycles between liquid N_2 and the water bath (55 ^oC) with 10 s of vortexing as the solution warmed up. The solution was left at each temperature extreme for 5 min. 1.2 mL of the lipid solution was then taken from the water bath at (60 ^oC), transferred into a 1 mL extrusion syringe, and set on an extrusion block and syringe/filter apparatus which had been pre-heated to 60 ^oC. The solution was extruded 23 times through a 400 nm filter. The equipment was rinsed with MeOH followed by 120 mM aqueous calcium acetate solution. The solution was then extruded 23 times through 200 nm filter and cooled to 4 ^oC for 10 min.

Gradient establishment: 0.95 mL of the liposome solution was recovered and transferred to a pre-prepared Float-a-Lyser G2 0.5-1kDa 1 mL dialysis tube, and the solution dialysed against 200 mL of 120 mM sodium sulphate (Na_2SO_4) solution (buffer preheated to 40 ^oC) for 1 hr. This was repeated twice more, using 200 mL of 120 mM sodium sulphate (Na_2SO_4) solution (buffer preheated to 40 ^oC). A fourth cycle of dialysis against 400 mL 120 mM sodium sulphate (Na_2SO_4) solution was conducted at ambient temperature for 12 h.

Drug loading: The liposome solution (0.95 mL) was transferred to a glass vial and 14 mg iproplatin was added. The solution was stirred gently at 55 ^oC for 1h. The solution was cooled to 4^oC and then columned on Sephadex columns (pre-equilibrated with 120 mM sodium sulphate (Na_2SO_4) solution) to remove unencapsulated drug. The pink band corresponding to the liposomes was collected; unencapsulated iproplatin was observed as a yellow band which followed the liposome band. A 200 µL aliquot of the sample was removed for digestion and analysis by ICP-MS.

Concentration determination of iproplatin in liposomes

Liposome sampling protocol: The liposomal solution (1 mL) was passed through a PD-50 Sephadex gel filtration column to remove unencapsulated iproplatin. Aliquots (2 x 70 µL) for ICP-MS were removed from the freshly purified sample (*see below*). Samples were taken immediately after initial purification, and then at regular timepoints, with results for the duplicate samples averaged over two (ICP-MS) injection replicates respectively. Liposomal solutions were stored at 4 ^{o}C between analyses.

Liposomal ICP-MS digestion protocol

Digest: Caution! Boiling Acid!

Romil ultrapure analytical grade (UPA) nitric acid was diluted with milliQ-filtered distilled water, to give a final concentration of 20% nitric acid stock solution. 10 ml volumetric flasks were cleaned overnight with 2 % nitric acid solution. The 70 µl liposome samples were briefly vortexed (30 s) before removing a 50 µL aliquot from each sample for analysis, to ensure homogeneity.

The 50 µl liposome sample was pipetted into a volumetric flask, followed by 450 µl of concentrated HNO_3 and 50 µl concentrated HCl. Then 100 µL H_2O_2 (30% solution) was added to each flask to aid digestion of organic components, and allowed to react until any foaming had ceased. Samples were heated on a stirrer-hotplate in a fume hood at 110 ^{o}C for 2 h, after which time the samples became transparent. The samples were allowed to cool to room temperature. If the samples were now shaken, no foaming was observed, indicating all organic compounds had been digested. If the sample foamed or was translucent, the samples were returned to heat and digestion was continued. When digestion was complete, distilled water was added to the volumetric flasks, accurately up to the 10 ml line. The samples were thoroughly homogenised by inverting, then 2 ml of each sample was removed, filtered (IM, PTFE) into falcon tubes, and further diluted with 8 ml distilled water. These samples were then diluted x 20 before ICP-MS to allow for elemental detection in the appropriate concentration range. Concentrations were determined for the ^{194}Pt and ^{195}Pt isotopes (and ^{31}P where relevant) and scaled according to isotopic abundance.

General microbubble (MB) Synthesis

A microbubble lipid film was prepared by dissolving DPPC and DSPE-PEG(2000) in 1 ml of chloroform in a 82:18 molar ratio, using 3.5 mg, 2.8 mg respectively, followed by solvent evaporation overnight on a 50 °C heat plate. The film was rehydrated in 2.5 ml of a (8:1:1 volume ratio) mixture of PBS, propylene glycol and glycerine for 60 min on a 50 °C heat plate. The solution was sonicated for 2.5 min at low power mode using a Microson XL-2000 (QSonica Ltd, Newtown, CT, USA). The lipid mixture was placed in an 0.5 ml Eppendorf and the headspace replaced with SF_6 (sulphur hexafluoride) gas. The solution was agitated for 60 s, using a Biosonics Capsule Mixer to produce microbubbles, which were then centrifuged and washed twice with PBS before being imaged by microscopy.

Production of coupled MB-L(Pt)

A microbubble lipid film was prepared by mixing dissolved DPPC, DSPE-mPEG(2000) and DSPE-PEG(2000)-DBCO in 1 ml of chloroform in a 82:9:9 molar ratio, using 3.5 mg, 1.35 mg and 1.45 mg respectively, followed by solvent evaporation overnight on a 50 °C heat plate. The film was rehydrated in 2.5 ml of a (8:1:1 volume ratio) mixture of PBS, propylene glycol and glycerine for 60 min on a 50 °C heat plate. A 0.1 ml aliquot of azide-functionalised liposomes prepared by Method 3 (with freeze-thaw step) **-** with equimolar DSPE-PEG(2000)-N_3 in place of DSPE-PEG(2000) - was added to 0.5 ml of hydrated microbubble solution in a 1 : 5 liposome: microbubble *v/v* ratio and the total volume made up to 1 ml with the 8:1:1 PBS: propylene glycol: glycerine solution. The mixture was agitated gently for 20 h to enable the azido-DBCO coupling to occur. The solution was sonicated for 2.5 min at low power mode using a Microson XL-2000 (QSonica Ltd, Newtown, CT, USA). For each sample, an aliquot of 250 µl of the microbubble lipid mixture was placed into 0.5 ml Eppendorf and the headspace replaced with SF_6 (sulphur hexafluoride) gas. The solution was agitated for 60s, using a Biosonics Capsule Mixer. Samples were centrifuged and washed twice with PBS to remove unbound liposomes.

Biological methods

MCF 7 breast cancer cells were cultured in RPMI medium supplemented with 10% FBS and 1% penicillin-streptomycin at 37 °C in 5% CO2 and 100% humidity.

MTT assays: were used to measure the viability of MCF7 cells after treatment. Following contact time with the sample and washing, an MTT solution in media was prepared to a concentration of 0.5 mg.ml^{-1} and added to cells. For IC_{50} experiments, 200 μl of MTT solution was added, and cells incubated for 2 h. For ultrasound experiments, each plate was incubated with 600 μl of solution for 2 h. Viable cells produced formazan crystals. The media was then discarded, and DMSO added to solubilize the formazan crystals formed (500 μl for ultrasound, 150 μl for IC_{50}). The absorbance of 100 μl of each sample, in duplicate, was measured in a 96-well plate at 540 nm (maximum absorbance of formazan product in DMSO) and 650 nm (reference absorbance to test for differences in the plastic), with 22 flashes per well and no temperature control.

Determination of IC_{50} (iproplatin, MCF7 cell line): MCF7 cells were seeded at 20,000 cells per well in a 96 well plate in a 37 °C cell culture incubator. After overnight incubation to allow attachment, the media was replaced with iproplatin supplemented media at different concentrations and returned to the incubator for 24 h. The drug was then removed from the cells by washing with PBS before an MTT assay was performed. Results are shown in Figure S8.

General Ultrasound Methods

Ultrasound setup: MCF7 cells were seeded in an Ibidi dish (10 ml) at a concentration of ~1-2x10^5 cells/dish and incubated at 37 °C in a humid, CO_2 incubator overnight, to allow cell attachment. Cells were washed once in serum free media (2 - 5 ml) to remove serum components. The Ibidi dish was half-filled with serum free media (5 ml) and 200 μl of sample added. Each plate was carried out in triplicate. The Ibidi dish was sealed with an acoustically transparent polydimethylsiloxane (PDMS) lid (Sonolid) and filled with serum free media via the entry hole on top, letting air out of the exit hole, before sealing with 6 mm PEEK rods. Samples were placed into the SAT2 ultrasound device (Figure 2) and treated with ultrasound for 3 min. Ultrasound parameters were set to - centre frequency: 1 MHz, acoustic pressure: 148 kPa (peak to peak), pulse repetition frequency: 100 Hz, duty cycle: 30%, pulse length: 3000 cycles, exposure time: 180 s. These parameters were selected based on previous experiments with this cell line and a similar microbubble preparation. Samples without ultrasound applied were loaded into the setup for 3 minutes (sham irradiation). Samples were returned to the incubator for 60 min, then washed 6 times in PBS to remove free iproplatin. Samples were then re-filled with culture media and incubated.

Ultrasound platinum cellular uptake experiments

Ultrasound and microbubble-mediated cellular accumulation of free iproplatin

Microbubbles (MB) were synthesised as described in the "General microbubble (MB) synthesis" section above. Experiments were conducted in triplicate. Solutions were made as follows: Samples **1** and **2**: 120 µl MB solution, 80 µl iproplatin solution, 50 µl PBS; Samples **3** and **4**: 170 µl PBS solution, 80 µl iproplatin solution. Following mixing, aliquots of 200 µl of these solutions were then added to the cells. This was followed by irradiation or sham irradiation of the cells for 3 min. Following treatment with ultrasound, cells were incubated for 60 min, washed with PBS (6x), followed by cell lysis with DMSO (500 µl) and collected to analyse by ICP-MS.

Ultrasound-mediated iproplatin release from L(Pt) and MB-L(Pt) and cellular accumulation

Microbubble-liposome drug delivery vehicle MB-L(Pt) was assembled as described in the *Production of coupled MB-L(Pt)* section above. Experiments were conducted in triplicate. Solutions were made as follows: Samples **1** and **2:** 200 µl MB-L(Pt) solution; Sample **3:** 200 µl liposome solution. This was followed by irradiation or sham irradiation of the cells for 3 min. Following treatment with ultrasound, cells were incubated for 60 min, washed with PBS (6x 5 ml), followed by cell lysis with DMSO (500 µl) and collected for analysis by ICP-MS.

Structures of lipids and cholesterol used

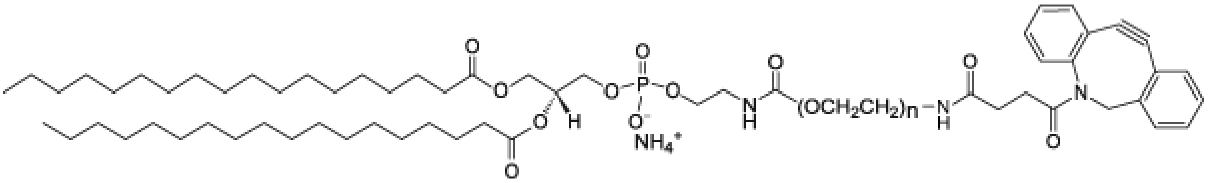

DSPE-PEG(2000)-DBCO (880229); MW = 3077.839

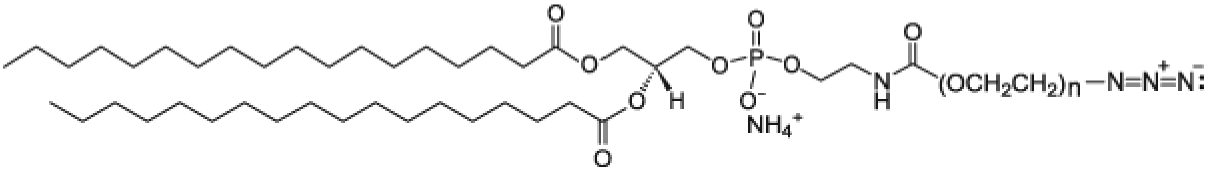

DSPE-PEG(2000)-Azide (880228); MW = 2816.519

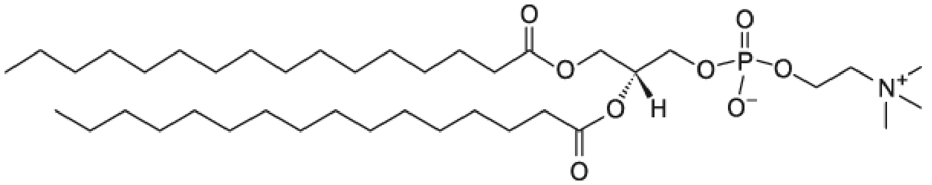

DPPC (850355); MW = 734.039

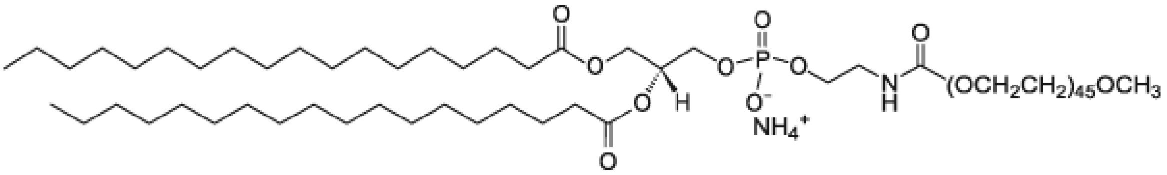

DSPE-PEG(2000) (880120); MW = 2805.497

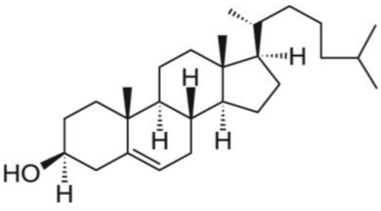

Cholesterol (700100); MW = 386.654

Figure S1. Structures of key lipids used in this study (with Avanti Polar product codes in brackets).

Stability of iproplatin in the presence of lipids

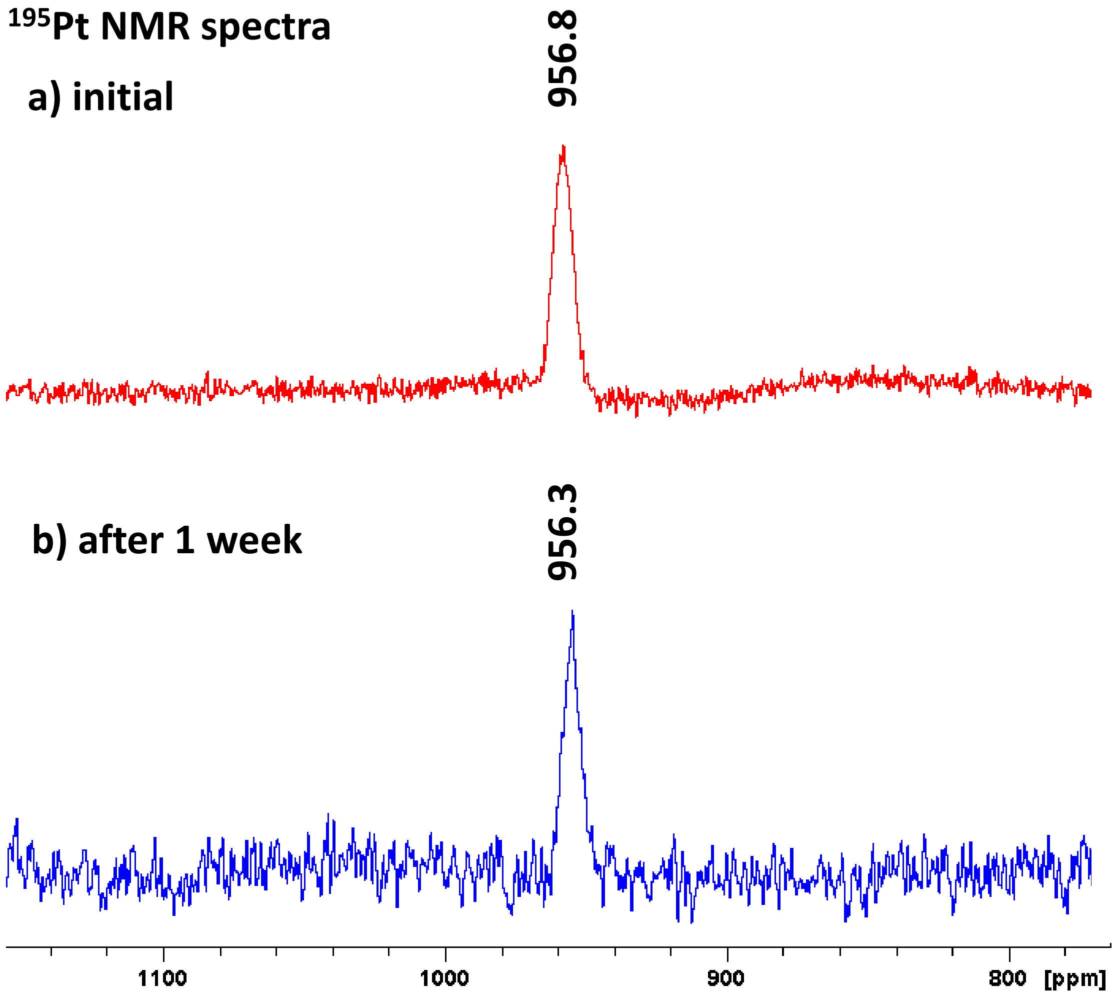

Figure S2. ^{195}Pt NMR spectra of iproplatin in the presence of lipids; (7 mg iproplatin in 0.4 ml of the unextruded stock solution of DPPC, cholesterol and DSEP-PEG(2000), diI dye in 120 mM [Ca(OAc)$_2$]; plus 0.1 ml D_2O); (a) immediately after mixing and (b) 1 week after mixing (storage at ambient temperature).

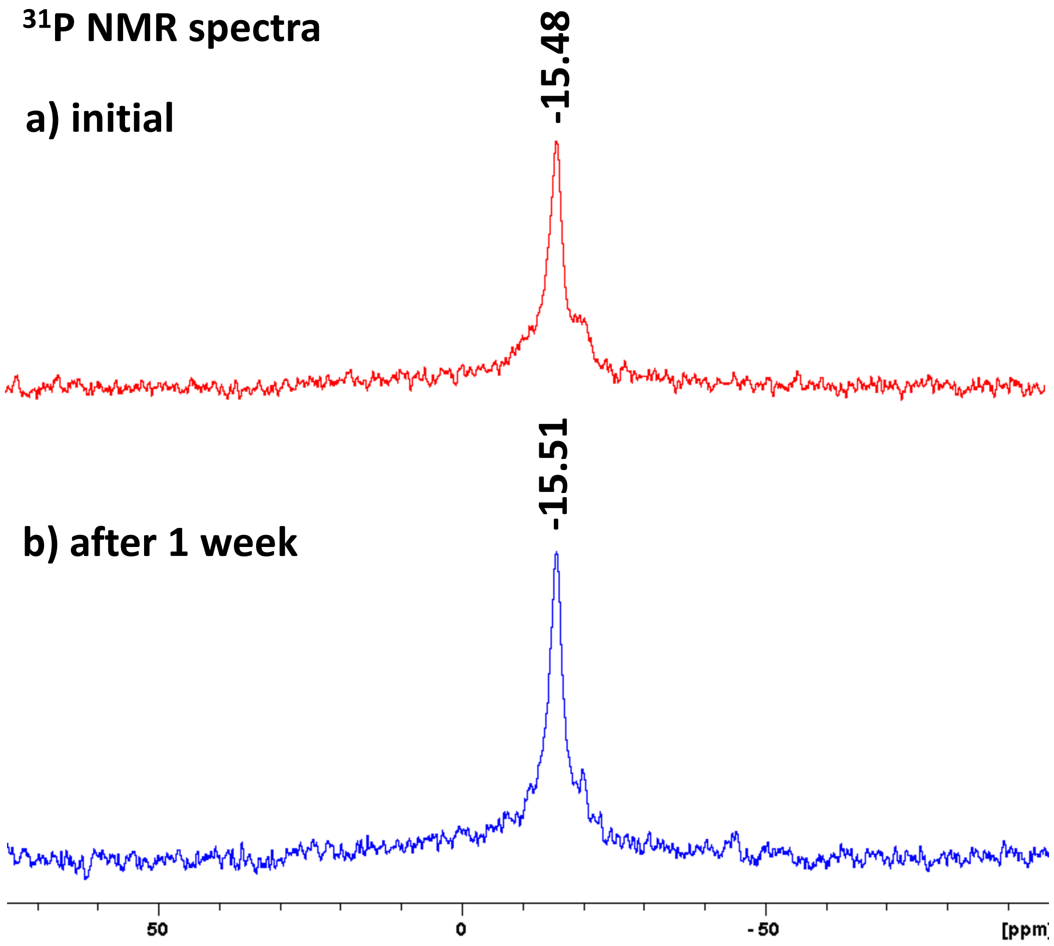

Figure S3. ^{31}P NMR spectra of iproplatin in the presence of lipids; (7 mg iproplatin in 0.4 ml of the unextruded stock solution of DPPC, cholesterol and DSEP-PEG(2000), diI dye in 120 mM [$Ca(OAc)_2$]; plus 0.1 ml D_2O); (a) immediately after mixing and (b) 1 week after mixing (storage at ambient temperature).

Stability of iproplatin to ultrasound

To test the stability of iproplatin when exposed to ultrasound irradiation, an experiment was conducted in which samples of iproplatin were analysed by analytical HPLC and ^{1}H NMR spectroscopy with and without exposure to sonication.

From a stock solution of 2 mL of a 3 mM solution of iproplatin in D_2O, 0.6 mL aliquots were taken. One to be analysed without sonication (negative control), and two to be analysed following sonication. For samples exposed to ultrasound, a QSonica probe sonicator (part no. Q700) was used (20 kHz output frequency). Samples were exposed to sonication for 3 minutes at 30% amplitude. No observable changes in ^{1}H NMR spectral resonances corresponding to iproplatin (^{1}H NMR 400 MHz, D_2O) δ: 3.27 (sept, 2H, CH), 1.30 (d, 12H, CH_3), 0.42 (br, 2H, OH), or reduction in intensity of ESI-MS signals corresponding to iproplatin species (*m/z*; 419.10 ($[M+H]^+$), 441.03 ($[M+Na]^+$); 837.12 ($[2M+H]^+$ 837.11) were observed between the negative controls and the irradiated samples, and no obvious new species were detected.

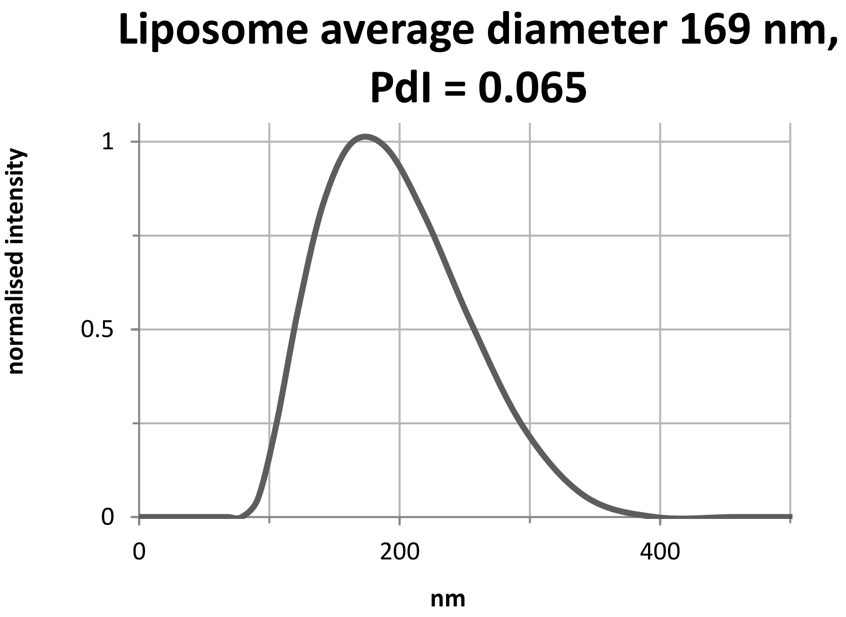

Figure S4. Dynamic light scattering (DLS) data for liposomes prepared according to method **1**, showing average diameter and polydispersity index (PdI), as an average of 3 measurements.

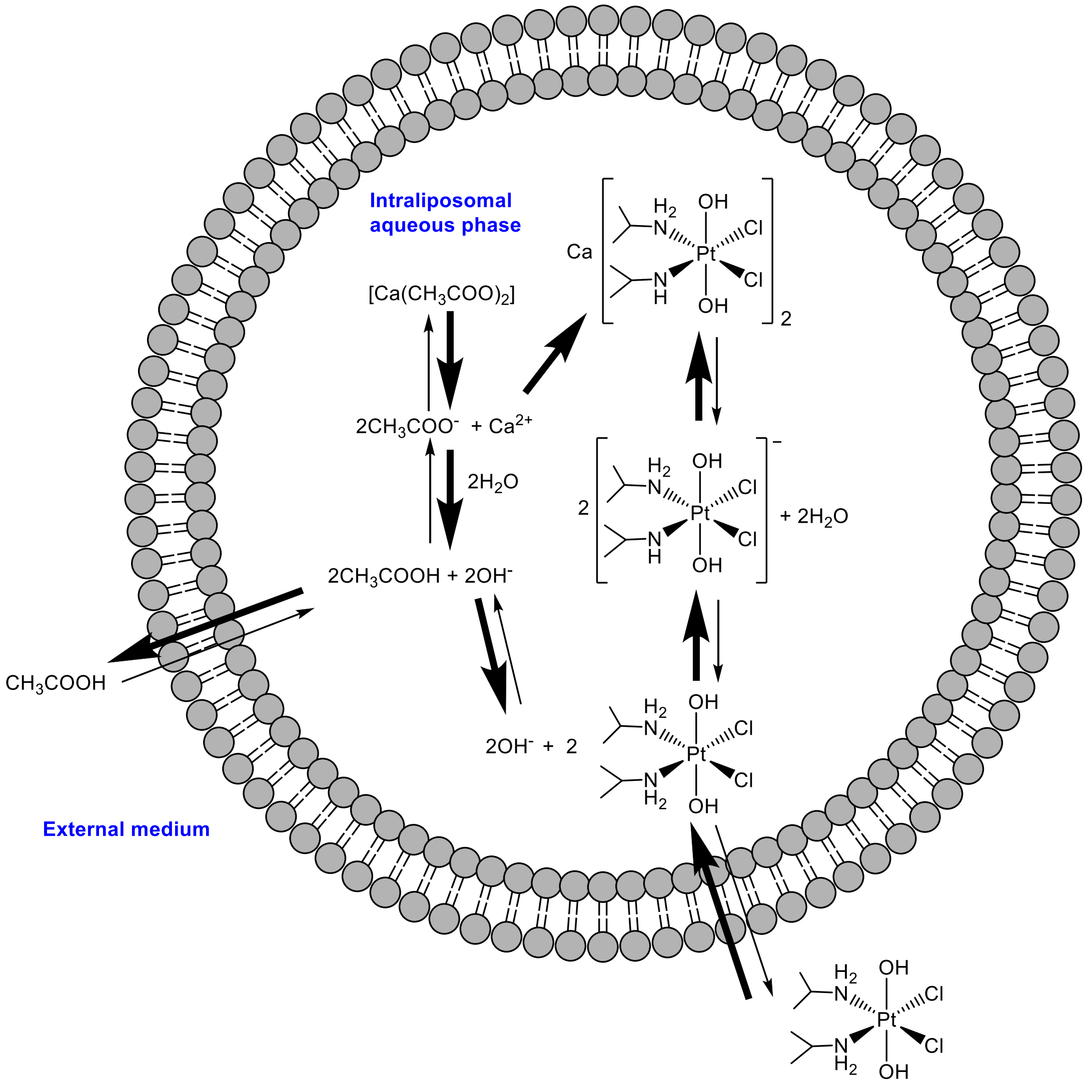

Figure S5. Proposed mechanism by which calcium acetate loading protocol enhances the liposomal loading of iproplatin (adapted from ref[5]).

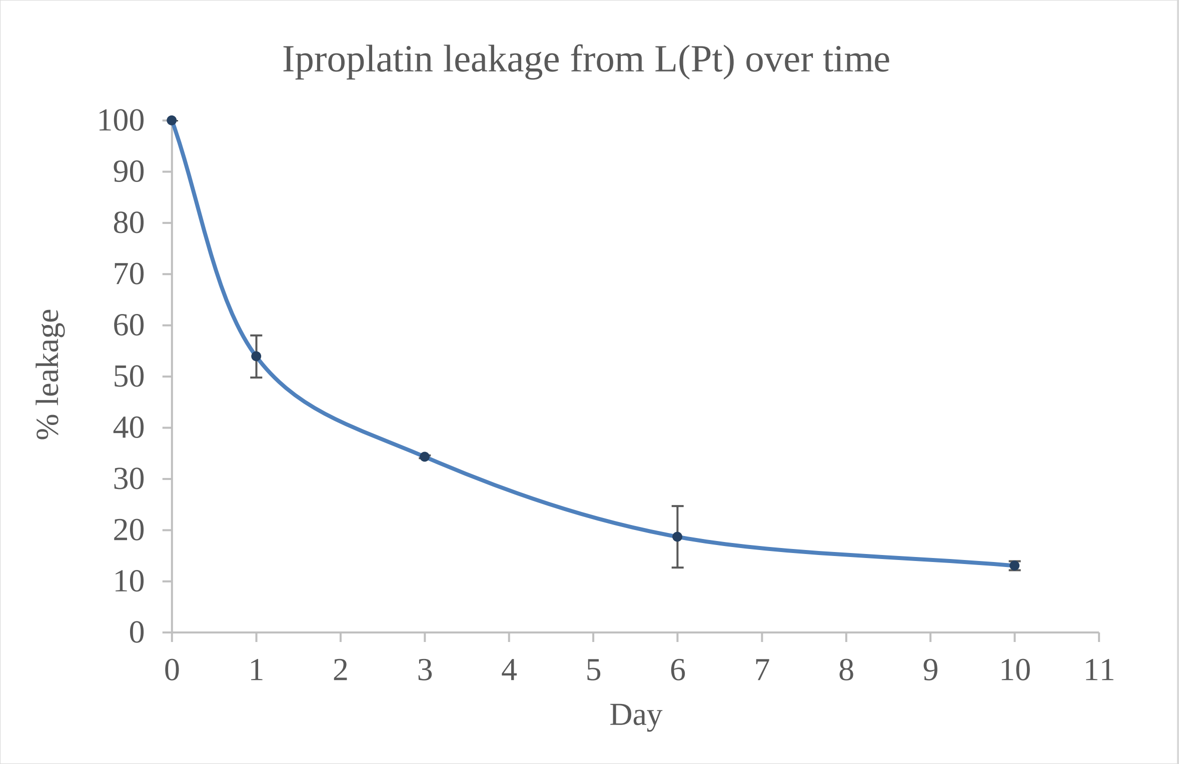

Figure S6. Passive release of iproplatin from liposomes (as determined by ICP-MS of Pt content) loaded by method **3** (calcium acetate loading), with liposomes stored at 4 °C between sampling points.

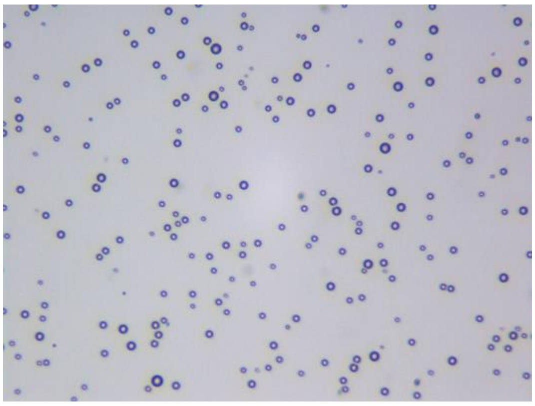

Brightfield image showing MB-L(Pt) constructs, X40 magnification.

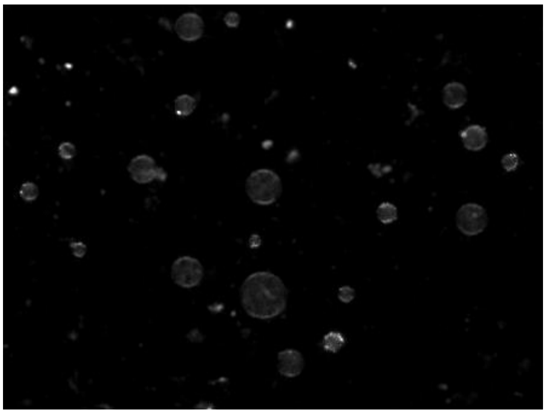

Fluorescence at diI output only, X40 magnification, post-wash.

Figure S7. Microscopy images: brightfield (*left*) and fluorescence (right) of MB-L(Pt) construct.

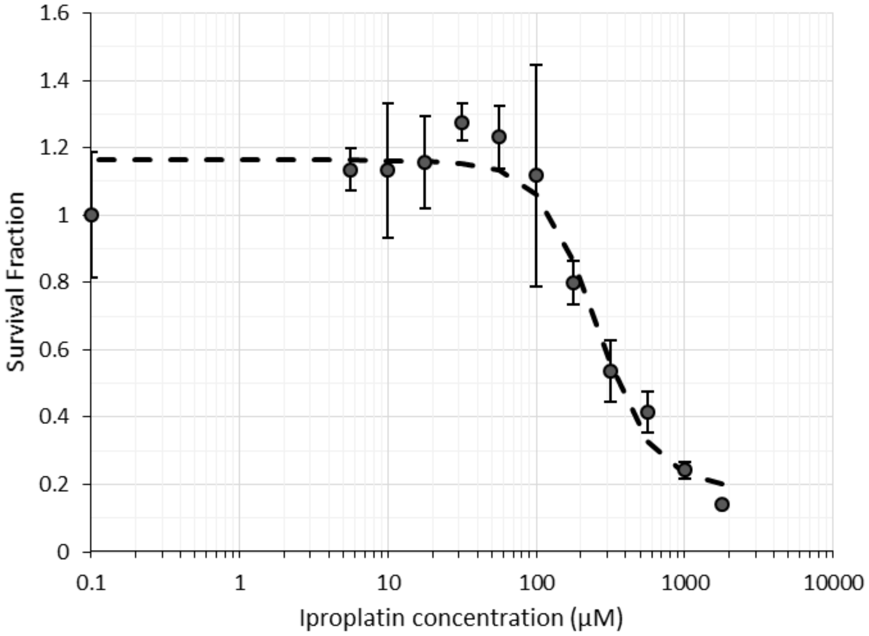

Figure S8. IC_{50} survival fraction of MCF7 cells treated for 24 h with iproplatin. After drug incubation, an MTT assay was performed with absorbance readings normalised to 0 µM iproplatin and plotted as points above. Due to log scaling on the concentration axis, the 0 µM result value is plotted as 0.1 µM. Best-fit values, Hill Slope constant (-2.281) and the IC_{50} value (254.6 µM) were calculated in PRISM 8.0 (GraphPad Software, San Diego, CA, USA) and used to plot the fitted curve (dashed line). Concentrations were tested in triplicate, error bars show standard deviation.

Iproplatin Calibration Curve for LCMS

Samples were prepared by adding one volume equivalent of MeCN to the aqueous iproplatin solution and sonicating for 2 min, before filtering (IM, Nylon) and LCMS analysis.

A calibration curve for iproplatin concentration was determined by LCMS analysis. ESI-MS of iproplatin [M] samples gives rise to two main species; $[M+H]^+$ (419.18 *m/z*) and $[2M+H]^+$ (837.20 *m/z*). Samples were analysed by measuring the intensity of the ESI-MS signal corresponding to the $[2M+H]^+$ peak, discounting the $[M+H]^+$ ion, which accounted for < 2% of the overall ion count of the injected samples at all concentration points. The LCMS spectrum was integrated over the mass range 830 – 845 *m/z*. A notable deterioration in the signal-to-noise ratio was observed in the LCMS at a concentration of 30 µM iproplatin, which was considered to be the limit of acceptable quantitation.

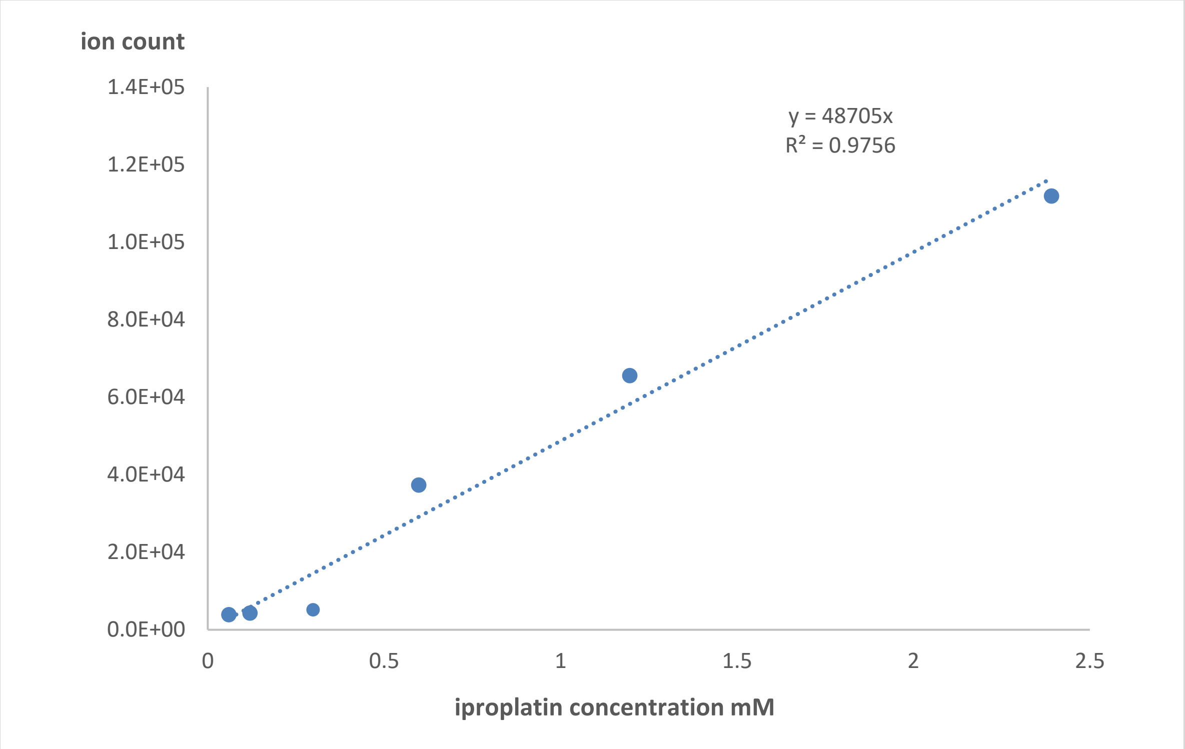

Figure S9. LCMS calibration: increasing concentrations of iproplatin with ion count corresponding to the $[2M+H]^+$ species at 837.20 *m/z*, with integration over the range 830 – 845 *m/z.*

References

[1] K. Matsuzaki, O. Murase, K. Sugishita, S. Yoneyama, K. Akada, M. Ueha, A. Nakamura, S. Kobayashi, *Biochim. Biophys. Acta* **2000**, *1467*, 219–226.

[2] https://avantilipids.com/divisions/equipment-products/mini-extruder-extrusion-technique/, *last accessed 26-09-19*.

[3] H. Jackson, C. A. McAyliffe, A. Perera, H. L. Sharma, N. J. Tinker, *J. Label. Compd. Radiopharm.* **1991**, *10*, 1121–1130.

[4] A. Schroeder, R. Honen, K. Turjeman, A. Gabizon, J. Kost, Y. Barenholz, *J. Control. Release* **2009**, *137*, 63–68.

[5] S. Clerc, Y. Barenholz, *Biochim. Biophys. Acta* **1995**, *1240*, 257–265.

[6] J. Gubernator, *Expert Opin. Drug Deliv.* **2011**, *8*, 565–580.

[7] X. Liu, P. Gong, P. Song, F. Xie, A. L. Miller II, S. Chen, L. Lu, *Biomater. Sci.* **2018**, 623–632.
